# Using Skewness and the First-Digit Phenomenon to Identify Dynamical Transitions in Cardiac Models

**DOI:** 10.3389/fphys.2015.00390

**Published:** 2016-01-11

**Authors:** Pavithraa Seenivasan, Soumya Easwaran, Seshan Sridhar, Sitabhra Sinha

**Affiliations:** ^1^Theoretical Physics Group, The Institute of Mathematical SciencesChennai, India; ^2^Scimergent Analytics and Education Pvt Ltd.Chennai, India

**Keywords:** cardiac arrhythmia, skewness, Benford's law, first-digit phenomenon, dynamical transitions, 87.19.Hh, 05.45.-a

## Abstract

Disruptions in the normal rhythmic functioning of the heart, termed as arrhythmia, often result from qualitative changes in the excitation dynamics of the organ. The transitions between different types of arrhythmia are accompanied by alterations in the spatiotemporal pattern of electrical activity that can be measured by observing the time-intervals between successive excitations of different regions of the cardiac tissue. Using biophysically detailed models of cardiac activity we show that the distribution of these time-intervals exhibit a systematic change in their skewness during such dynamical transitions. Further, the leading digits of the normalized intervals appear to fit Benford's law better at these transition points. This raises the possibility of using these observations to design a clinical indicator for identifying changes in the nature of arrhythmia. More importantly, our results reveal an intriguing relation between the changing skewness of a distribution and its agreement with Benford's law, both of which have been independently proposed earlier as indicators of regime shift in dynamical systems.

## 1. Introduction

Many vital physiological processes are characterized by rhythmic activity, ranging from the circadian clock regulating the daily sleep-wake cycle to temporal patterns of respiration that occur over a scale of seconds (Glass, 2001). The periodic beating of the heart, that results in constant circulation of oxygenated blood throughout the body, is one of the most important of such naturally occurring oscillatory phenomena in the body (Zipes and Jalife, 2013). Certain types of disturbances in the cardiac rhythmicity, referred to as arrhythmia, can severely impair the normal functioning of the heart and in the most critical instances, can result in sudden cardiac death (Winfree, 1980). Such “dynamical diseases” (Mackey and Glass, 1977; Belair et al., 1995), i.e., diseases resulting from abnormal activity in an otherwise intact physiological system, are a significant public health burden in developed countries. For example, in the United States, diseases of the heart constitute the leading cause of death (responsible for about 25% of all deaths), of which more than half can be classified as sudden cardiac deaths (Hennekens, 1998; Zheng et al., 2001; Heron, 2013). Even in developing countries, in recent times heart disease has overtaken other causes of death, e.g., sudden cardiac deaths contributed to about 10% of overall mortality in certain regions in India, accounting for upto half of all cardiovascular-related deaths (Madhavan et al., 2011; Rao et al., 2012).

Several studies have shown that early detection of onset of arrhythmia resulting in prompt therapeutic intervention significantly improves the chances of surviving such episodes (Gold et al., 2010; Travers et al., 2010). Thus, developing methods for identifying signs of impending arrhythmic events with potentially serious consequences can significantly contribute toward reducing the mortality rate due to sudden cardiac death. With this aim in view there have been a number of attempts at applying time-series analysis methods on cardiac activity data in order to extract robust indicators of imminent instances of temporal irregularities in the heart. However, the complexity of heart rate dynamics makes it difficult to characterize and distinguish the temporal signatures of a healthy heart from a diseased one (Christini et al., 1995; Kurths et al., 1995; Cohen and Taylor, 2002; Goldberger et al., 2002; Voss et al., 2009; Shiogai et al., 2010; Iatsenko et al., 2013).

Most studies of cardiac time-series have focused on heart rate variability as measured by temporal changes in the *R-R interval*, the duration between successive episodes of ventricular depolarization which triggers contraction of the lower chambers of the heart. Following the pioneering observations connecting decreased variance in R-R intervals with higher mortality risk in patients suffering myocardial infarction (Wolf et al., 1978; Kleiger et al., 1987), it is now generally accepted that healthy individuals have higher heart-rate variability compared to those with diseased hearts (Lombardi, 2000). However, certain pathological conditions including cardiac arrhythmia are seen to be extremely irregular (Costa et al., 2002). In fact, it has been observed that a transition from tachycardia, i.e., abnormally rapid heart-rate, to fibrillation, characterized by erratic muscle activity that prevents the heart from pumping blood, is marked by a switch from relatively more periodic activity to a highly irregular dynamical state (Garfinkel et al., 1997). While the R-R intervals in normal sinus rhythm appear to have almost as unpredictable a nature as that seen during fibrillation (Small et al., 2002), it has been suggested that the “chaoticity” during normal cardiac activity arises through interaction of the heart with the nervous system (Nakai et al., 2010). In contrast, the spatiotemporal chaos associated with fibrillation arises from intrinsic instabilities in cardiac excitation dynamics (Weiss et al., 1999).

One possible route from tachycardia to fibrillation that has been established through extensive simulation studies of models of cardiac electrical activity is the degeneration of reentrant spiral wave (corresponding to tachycardia) to disordered, turbulent activity (characterizing fibrillation) through spiral breakup (Fenton et al., 2002; Sridhar et al., 2013). This dynamical transition has been reproduced in a wide range of systems, from simple, excitable media to biologically detailed models, underlining the robustness of the scenario (Sinha and Sridhar, 2015). Thus, the study of spatiotemporal dynamics in models of electrical activity in cardiac tissue provides another perspective to identify indicators for an impending onset of possibly life-threatening arrhythmia.

In this paper, we focus on analyzing time-series data obtained from spatially extended models of cardiac ventricular activity in which, by tuning specific physiological parameters, one can observe transitions to different dynamical regimes representing various classes of arrhythmia. This allows us to look for statistical signatures that can help in early detection of arrhythmic episodes, where the observed patterns are exclusively due to abnormal excitation activity that characterizes such arrhythmia and unrelated to heart rate variability that arises from the influence of the nervous system on the sinus node, the natural pacemaker of the heart (Lombardi and Stein, 2011). This study, therefore, provides a benchmark against which analysis of ECG data obtained from clinical studies can be compared, enabling distinction of statistical features of arrhythmic time-series that are intrinsic to the dynamics of heart muscle from those that are a result of changes in the autonomic modulation of cardiovascular function (achieved through dynamical balance between sympathetic and parasympathetic effects Purves et al., 2001). As signature patterns (if they exist) that indicate transitions from one dynamical regime of cardiac activity to another may be masked by other effects in reality, establishing them through analysis of the model output will allow us to look for them in data obtained from experimental or clinical studies.

**Figure 1:**
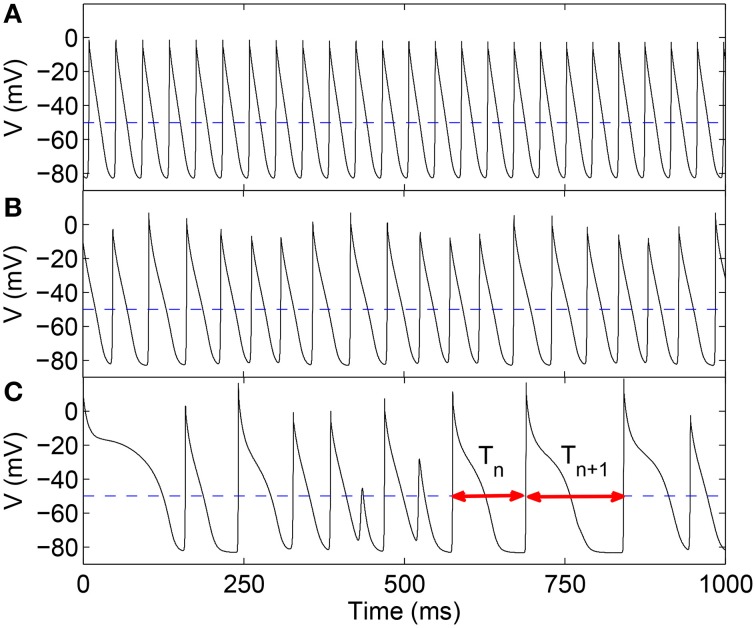
**Time-series of the transmembrane potential ***V*** representing local excitation activity in a two-dimensional LR1 model (***L*** = 400) for different values of the maximum Ca^**2+**^ channel conductance ***G***_***si***_, viz., (A) 0.005, (B) 0.04, and (C) 0.065 mS cm^**−2**^**. The distinct nature of the corresponding spatiotemporal dynamics, viz., rigid rotation of a spiral similar to monomorphic tachycardia **(A)**, chaotic meandering of spiral core representing polymorphic tachycardia **(B)**, and spatiotemporal chaos indicating fibrillation **(C)**, is visually apparent in the varying pattern of intervals between successive excitations. It is quantified in terms of the sequence of time-intervals {*T*_*n*_} (*n* = 1, 2, …) between each pair of consecutive local supra-threshold depolarizations (two such intervals are shown in **C** using double-headed arrows). We consider a local supra-threshold excitation event to have occurred when *V* exceeds −50 mV (broken line).

Here, for our statistical analysis, we have focused on the sequence of time-intervals *T* between successive excitations of ventricular muscle cells (analogous to the R-R interval for ECGs) as a representative feature of heart rate dynamics (Figure 1). An important result of our study is that the distribution of these intervals exhibit clearly observable changes in their moments—in particular, the skewness—around the onset of qualitatively distinct dynamical behavior characterizing various arrhythmic episodes (represented by the different panels in Figure 1). Intriguingly, we also observe that at these transitions, the distribution of the time-intervals appears to agree better with Benford's law (BL), an empirically established feature of the frequency distribution of leading digits of numbers occurring in many phenomena in various physical, biological and social contexts (Nigrini, 1996; Hill, 1998; Sambridge et al., 2010; Friar et al., 2012). Both variation in skewness (Guttal and Jayaprakash, 2008; Scheffer et al., 2009) and closer agreement with BL (De and Sen, 2011) have independently been suggested as indicators of regime shifts or phase transitions in different systems. Our work not only finds both of these signatures to be indicative of the onset of arrhythmic behavior, but additionally suggests that these two phenomena (viz., increasing skewness and agreement with BL) may be related.

## 2. Materials and methods

### 2.1. Model

To simulate spatiotemporal excitation activity in cardiac muscle under different physiological conditions, we have used a two-dimensional model of ventricular tissue having the generic form

(1) $$\begin{array}{l} \frac{\partial V}{\partial t}=\frac{-I_{ion}\left(V,g_{i}\right)}{C_{m}}+D\nabla^{2}V, \end{array}$$

where *V* (mV) is the potential difference across a cellular membrane, $C_{m}\left(=1\mu \text{F}{\text{cm}}^{-2}\right)$ is the transmembrane capacitance, *D* is the diffusion constant (= 0.001cm^2^s^−1^ for the results reported in the paper), $I_{ion}\left(\mu {\text{Acm}}^{-2}\right)$ is the total current density through ion channels on the cellular membrane, and *g*_*i*_ describes the dynamics of gating variables of different ion channels. The specific functional form for *I*_*ion*_ that we have focused on here is that of the Luo-Rudy I (LR1) model which describes the ionic currents in a guinea pig ventricular cell (Luo and Rudy, 1991):

$$\begin{array}{l} I_{ion}=I_{Na}+I_{K}+I_{K1}+I_{Kp}+I_{si}+I_{b}, \end{array}$$

where $I_{Na}=G_{Na}m^{3}hj\left(V-54.4\right)$ is the fast inward Na^+^ current, *I*_*si*_ = *G*_*si*_*df*(*V* − *E*_*si*_) is the slow inward Ca^2+^ current where *E*_*si*_ = 7.7−13.0287 ln ([Ca^2+^]_*i*_) is the reversal potential, dependent on the intracellular ion concentration [Ca^2+^], *I*_*K*_ = *G*_*K*_*xx*_1_(*V* + 77.62), *I*_*K*1_ = *G*_*K*1_*K*1_∞_(*V* + 87.95), and *I*_*Kp*_ = 0.0183*Kp*(*V* + 87.95) are three different types of K^+^ current, and *I*_*b*_ = 0.03921(*V* + 59.87) is a background current. The currents are determined by ion channel gating variables *m*, *h*, *d*, *f* and *x*, whose time evolution is described by ordinary differential equations of the form, *dϵ*∕*dt* = (ϵ_∞_ − ϵ)∕τ_ϵ_, where ϵ_∞_ is the steady state value of ϵ (= *m*, *h*, *j*, *d*, *f*, and *x*) and τ_ϵ_ is the corresponding time constant obtained by fitting experimental data. Parameter values used are as in Luo and Rudy (1991), except for *G*_*K*_ which is set to 0.705 mS/μF and *G*_*si*_ that is varied to alter the stability of spiral wave dynamics (Xie et al., 2001). We have explicitly verified that our results are not sensitively dependent on model-specific details, viz., the description of ion-channel dynamics, by observing qualitatively similar behavior with another functional form for *I*_*ion*_ described in the ten Tusscher-Panfilov (TP06) model of a human ventricular cell (ten Tusscher and Panfilov, 2006).

For numerical simulations, the two-dimensional spatially extended system is discretized on a grid of size *L* × *L* (= 400 for LR1 model and 1024 for TP06 model) with a space step of δ*x* (= 0.0225 cm for LR1 model and 0.025 cm for TP06 model). The equations are solved using a forward Euler method with time step δ*t* (= 0.05 ms for LR1 model and 0.02 ms for TP06 model) and a standard 5-point stencil for the Laplacian describing the spatial coupling between the lattice elements. No-flux boundary conditions are implemented at the edges. The initial spiral wave state is obtained by generating a broken wave front which then dynamically evolves into a curved rotating wave. The movement of the spiral wave core is obtained by tracing the trajectory of intersection points of iso-contour lines for a pair of dynamical variables of the model, viz., *V* and *h* in the LR1 model (Barkley et al., 1990).

### 2.2. Inter-spike interval time-series

We analyze the statistical properties of the time-intervals between successive excitations (i.e., depolarization) at specific locations in the simulated cardiac tissue. Each point in the simulation domain is considered to be excited if the corresponding transmembrane potential *V* crosses a threshold value (set equal to −50 mV here, although our results are robust with respect to the choice of threshold) from below, i.e., from a hyperpolarized state. The time-interval *T* between two such successive crossings of the threshold is recorded for constructing the data-set, values being sampled from 400 equally spaced points arranged in a regular grid on the simulation domain (for the LR1 model). Typically time series of 5 s total duration were used for our analysis. From the data-sets obtained at different values of *G*_*si*_, the corresponding distributions for *T* are obtained and the moments calculated, including mean μ, standard deviation σ, and skewness, the latter being measured by the Pearson's moment coefficient of skewness defined as γ = *E*[(*X* − μ)∕σ)^3^]. Using other measures for the skewness did not qualitatively alter our results. The time-interval distributions obtained for different parameters are also tested for the degree of agreement with Benford's Law.

### 2.3. BL and benford distribution

Named after the American physicist F. Benford who made the first-digit phenomenon widely known, BL had been noticed in numbers associated with a variety of natural (as well as social) phenomena as far back as in 1881 by the astronomer S. Newcomb. According to this empirical law, numbers beginning with 1 or 2 occur more often than those beginning with 8 or 9 (Fewster, 2009). Specifically, the probability of the first or leading digit of such numbers being *i* (*i* = 1, 2, …9) is given by the *Benford distribution*:

$$\begin{array}{l} P\left(i\right)=\log_{10}\left(1+\frac{1}{i}\right). \end{array}$$

The reason for the ubiquity of this distribution has been connected to its scale-invariance and base-invariance (Hill, 1995). Thus, if indeed there is a universal principle underlying the distribution of the leading digits of numbers which is independent of the units in which the numbers are measured or the number base used, then the BL follows. Simple mathematical arguments have been used to show that any distribution of numbers arising from natural processes that spans several orders of magnitude and is reasonably smooth will obey BL (Fewster, 2009). The Benford distribution, as mentioned earlier, is seen in many empirical data-sets, including those arising in a biological context, such as, the distribution of open reading frames in prokaryotic and eukaryotic genomes (Friar et al., 2012). Dynamical systems, such as those describing the molecular dynamics of fluids or certain chaotic systems, also exhibit BL in the numbers expressing coordinates of the generated trajectories (Tolle et al., 2000; Snyder et al., 2001). More recently, BL has been used as a signature for detecting phase transitions in a quantum system (De and Sen, 2011).

In order to compare the distribution of the intervals *T* between successive excitations with that expected from BL, we first obtain a set of normalized time-intervals *t* by subtracting the minimum value of the series from all intervals *T* and dividing by the range, i.e., *t* = [*T*−min(*T*)]/[max(*T*)−min(*T*)]. The leading digits *i* of the normalized intervals *t* are then extracted as $i=\left\lfloor |t|∕10^{\left\lfloor \log_{10}\left(\left|t\right|\right)\right\rfloor }\right\rfloor$, where |*z*| and ⌊*z*⌋ indicate the absolute value of *z* and the largest integer not greater than *z*, respectively. The distribution of *i* is then tested for agreement with BL using statistical tests for goodness of fit.

### 2.4. Statistical tests for goodness of fit with BL

The goodness of fit between the two distributions (the empirical and that predicted by BL) is measured by a two-sample Kolmogorov-Smirnov test. We have used the function *kstest*2 implemented in *MATLAB* which returns a test decision for the null hypothesis that both the sets are from the same distribution, along with a *p*-value and the KS test statistic *k* describing the degree of deviation from BL. In our study the test statistic is the comparison parameter

$$k=\underset{i}{\max}\left(F_{c}\left(i\right)-B_{c}\left(i\right)\right),$$

which measures the maximum distance between the two cumulative distributions, *F*_*c*_(*i*) and *B*_*c*_(*i*), of the leading digits *i* of normalized time intervals and that expected from BL, respectively. A lower value of *k* implies closer agreement with the Benford distribution.

Apart from the KS test, we have also used the Pearson's chi-squared test to confirm the compliance of the empirical distributions with BL. The test statistic

$$\chi^{2}=\overset{n}{\underset{j=1}{\sum }}\frac{{\left(F_{j}-B_{j}\right)}^{2}}{B_{j}},$$

quantifies the total magnitude (over all *n* entries of the empirical time-series) of the difference between the two probability distributions, *F* and *B*, for the leading digits of the normalized time intervals and that expected from BL, respectively. As for the KS test, a lower value of χ^2^ implies closer agreement with the Benford distribution.

## 3. Results

To identify the statistical signatures characterizing dynamical transitions to different types of arrhythmia, we systematically explore the spatiotemporal dynamics of the model systems in different parameter regimes. The nature of the excitation activity is varied in a controlled manner by changing the kinetic properties of an ion channel, viz., increasing the maximum Ca^2+^ channel conductance *G*_*si*_ for the LR1 model (keeping all other model parameters unchanged) which is known to result in a succession of dynamical transitions (Qu et al., 2000) as seen in Figure 1. For the TP06 model, the maximum conductance *G*_*pCa*_ of the sarcolemmal pump current *I*_*pCa*_ is increased that eventually results in spiral breakup leading to spatiotemporal chaos (ten Tusscher and Panfilov, 2006).

**Figure 2:**
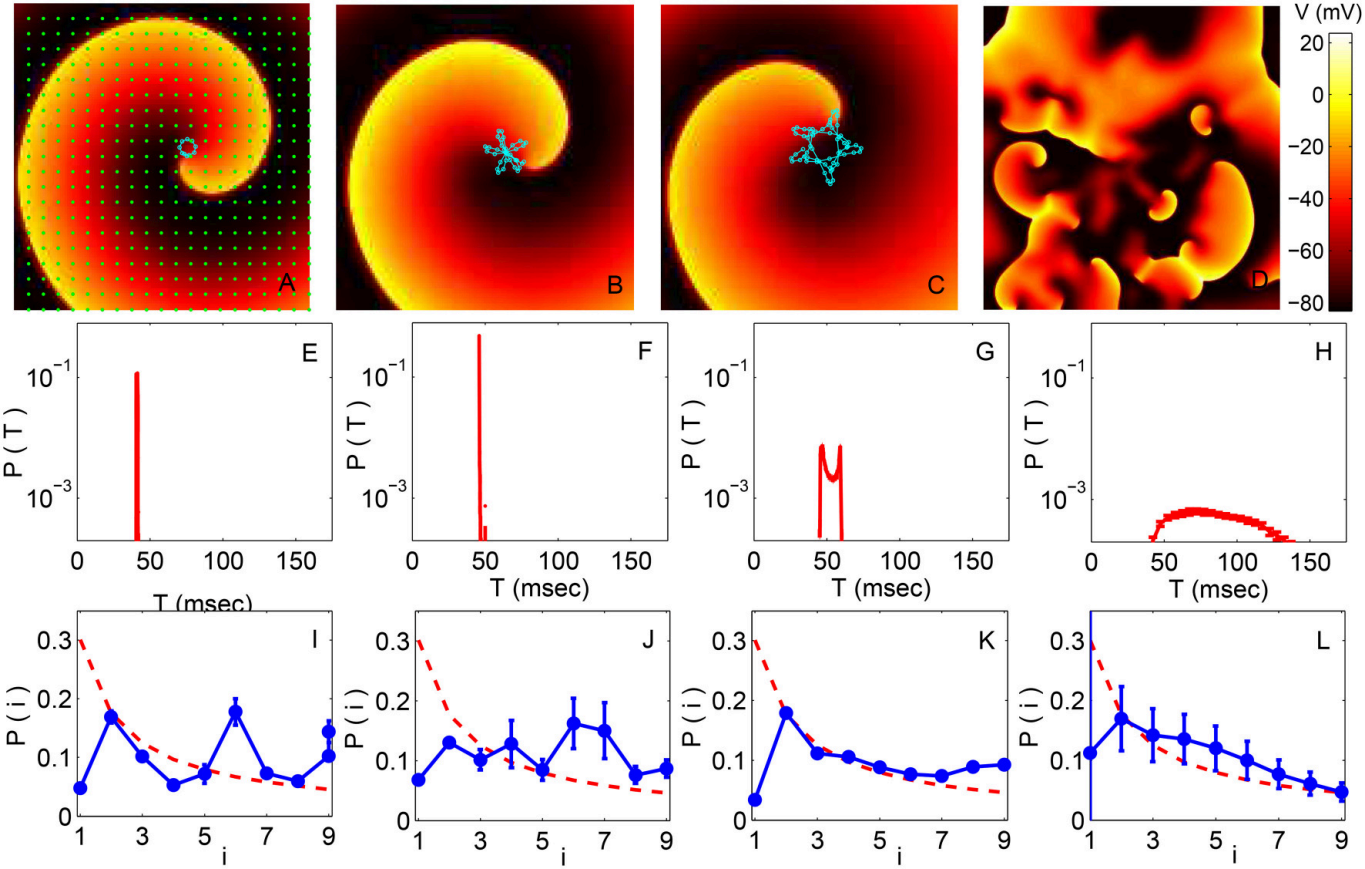
**(A–D)** Pseudocolor images of spatiotemporal activity (measured in terms of transmembrane potential *V*) for the two-dimensional LR1 model (*L* = 400) showing the different dynamical regimes obtained by increasing the maximum Ca^2+^ channel conductance *G*_*si*_ (expressed in units of mS cm^−2^). The successive panels represent a spiral wave undergoing **(A)** stable rotation (*G*_*si*_ = 0.005), **(B)** quasiperiodic meandering (*G*_*si*_ = 0.025), and **(C)** chaotic meandering (*G*_*si*_ = 0.04). Further increase of *G*_*si*_ results in breakup of the spiral wave leading to **(D)** spatiotemporal chaos (*G*_*si*_ = 0.065). The trajectory of the spiral core (the tip of the spiral wave, defined to be a phase singularity) for a duration of 500 ms is indicated in all panels except for the one corresponding to chaotic activity where there is a large multiplicity of singularities. **(E–H)** The probability distribution of time intervals *T* between successive supra-threshold activations of a local region corresponding to the dynamical regimes indicated in **(A–D)**, respectively. **(I–L)** Probability distribution of the leading digits *i* of the normalized time intervals between successive supra-threshold activations of a local region corresponding to the dynamical regimes indicated in **(A–D)**, respectively. The broken curve indicates the distribution predicted by Benford's law. Each distribution in **(E–L)** is obtained by averaging over data collected from many spatial positions in the simulation domain (indicated by points in **A**) and also over several realizations, with error bars indicating the standard deviation.

Representative images of the spatiotemporal dynamics (with LR1 model ion-channel kinetics) in the different regimes are shown in the top row of Figure 2, with the trajectory of the spiral core (traced in the first three panels using a light color) exhibiting characteristic changes in its qualitative nature. Starting from an initial state characterized by a rotating spiral wave, for small values of the conductance (e.g., *G*_*si*_ = 0.005) we observe rigid rotation with the core moving around an approximately circular trajectory (Figure 2A), which corresponds to the clinical phenomenon of monomorphic tachycardia. This gives way to meandering at higher values of *G*_*si*_ (≃0.025, see Figure 2B), followed by the appearance of chaotic meandering around *G*_*si*_ = 0.04 (Figure 2C) and finally the breakup of spiral waves leading to spatiotemporal chaos, representative of fibrillation, for values of *G*_*si*_ > 0.055 (Figure 2D).

The panels in the middle row of Figure 2 show the probability distribution of time intervals between successive excitations, *T*, for the *G*_*si*_ values corresponding to the panels in the top row. We observe that the range of these intervals become broader at larger *G*_*si*_ values as the dynamics of the spiral wave becomes more complex. A very narrow range of intervals is dominant at low *G*_*si*_, as expected for a rigidly rotating spiral wave having a characteristic period of rotation (Figure 2E). With increased meandering of the core, however, the time interval between successive excitations of a local region becomes more irregular, which is manifested as a broader distribution of *T* (Figure 2F). As the spiral core trajectory becomes even more complex, covering a larger portion of the simulation domain, we see that the distribution not only widens further but also develops multiple peaks at the extremities (Figure 2G). Finally, following breakup and spatiotemporal chaos, the time between successive excitations become essentially random in character with a distribution that spans a relatively large range of *T* (Figure 2H).

To see how closely the dynamical process follows the Benford distribution in the different regimes, in the bottom row of Figure 2 we show the probability distributions of the leading digits *i* of the normalized intervals *t*. It is evident that the distribution of *i* moves closer to the form expected for the Benford distribution (indicated by a broken curve) at larger values of *G*_*si*_. In fact, the empirical distribution shows the best agreement with BL in the spatiotemporally chaotic state corresponding to *G*_*si*_ = 0.065 (Figure 2L), which is consistent with the corresponding time interval distribution being exponential in nature - as it is known that values distributed exponentially follow BL. We see that that this distribution of leading digits *i* is closest to BL at the transition points corresponding to chaotic meandering (*G*_*si*_ = 0.040) and spiral breakup (*G*_*si*_ = 0.065).

**Figure 3:**
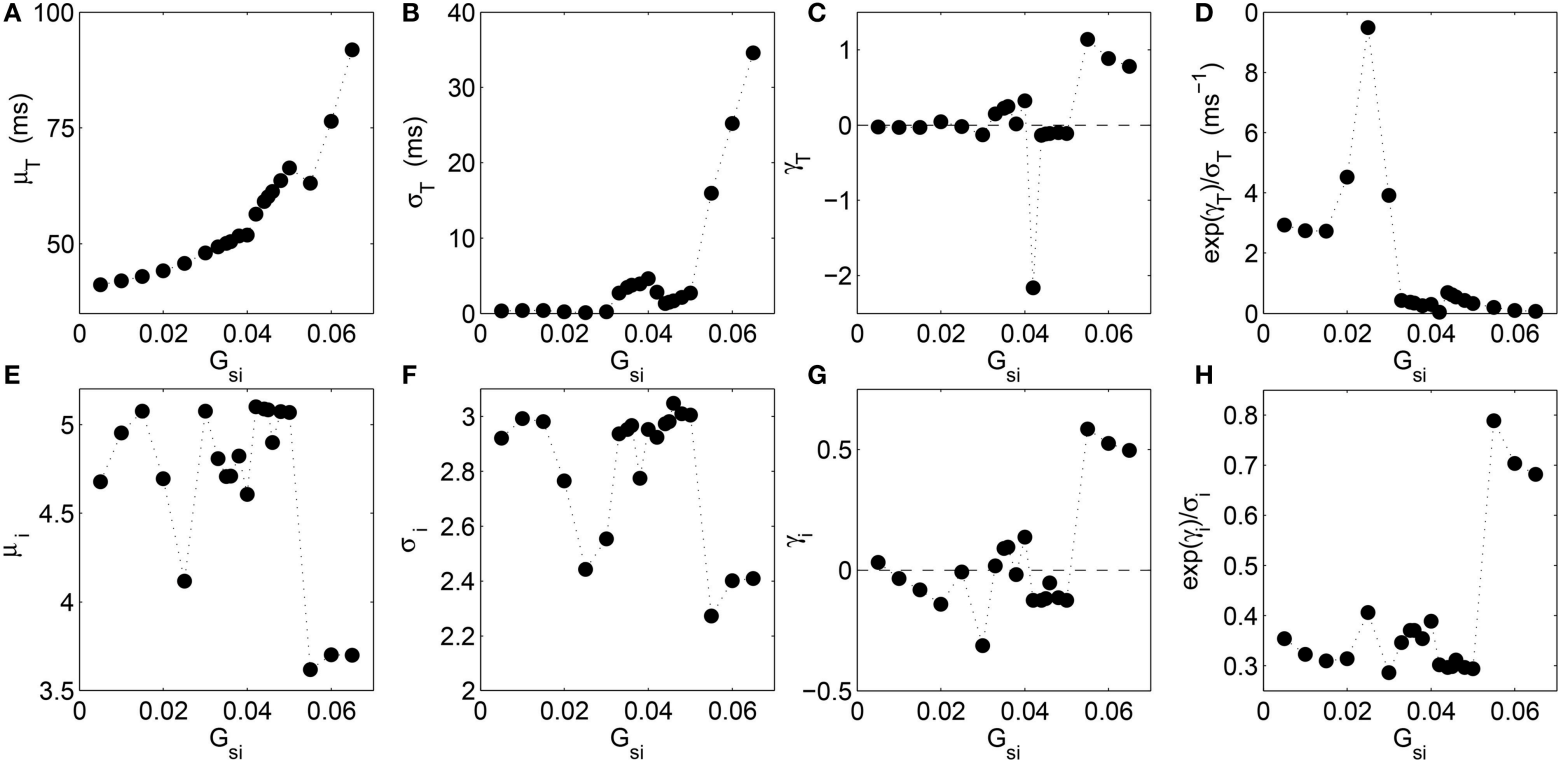
**Analysis of various moments for the distributions of the time intervals ***T*** between successive supra-threshold activations of a local region (A–D) and that of the leading digits ***i*** of the normalized time intervals (E–H) as a function of the maximum Ca^**2+**^ channel conductance ***G***_***si***_ in the two-dimensional LR1 model**. Variation in **(A)** the mean μ_*T*_, **(B)** standard deviation σ_*T*_, **(C)** skewness γ_*T*_ measured in terms of the Pearson's moment coefficient, and **(D)** the derived quantity *exp*(γ_*T*_)∕σ_*T*_, correspond to the distribution of the time intervals *T*, while the variation shown for **(E)** the mean μ_*i*_, **(F)** standard deviation σ_*i*_, **(G)** skewness γ_*i*_ (again measured in terms of the Pearson's moment coefficient), and **(H)** the quantity *exp*(γ_*i*_)∕σ_*i*_, correspond to the distribution of the leading digits *i*. Large changes in both the skewness measures (γ_*T*_ and γ_*i*_), and to an extent, the dispersion measures (σ_*T*_ and σ_*i*_) correspond to successive dynamical transitions between rigid rotation, quasiperiodic meander and chaotic meander of the spiral core, finally giving rise to spatiotemporal chaos resulting from spiral breakup. The measure *exp*(γ_*i*_)∕σ_*i*_ combines the information obtained from the variation of standard deviation and skewness, enabling it to indicate some of the dynamical transitions more clearly. The linear correlation coefficient between the skewness of *T* and that of *i* is *r*_γ_ = 0.67 (*p* = 0.001).

To understand the nature of the distributions in the different dynamical regimes better, we show how the moments of the distribution for the time intervals *T* and that for the corresponding leading digits *i* of the normalized intervals vary with increasing *G*_*si*_ (Figure 3). We observe that the mean value of the interval between successive excitations steadily rises with *G*_*si*_ as the complexity of the spiral core trajectory increases excepting for a small dip around *G*_*si*_ = 0.055 which is the point of transition to spiral breakup (Figure 3A). The dispersion of the *T* distribution, measured by its standard deviation σ_*T*_ (Figure 3B) shows a similar increasing nature with *G*_*si*_ although, around *G*_*si*_ = 0.04, where a transition occurs from quasiperiodic to chaotic meandering of the spiral core, there is a small decrease. The skewness γ of the distribution is the most informative of all the moments considered here, as it shows large deviations from zero only around critical values of *G*_*si*_ associated with transitions between different dynamical regimes. In particular, we notice peaks in the skewness at *G*_*si*_ = 0.025, 0.04, and 0.055 which correspond to transition to quasiperiodic meandering, chaotic meandering and spiral breakup, respectively (Figure 3C). In order to make the relation between the different moments and the dynamical transitions even more clear, we have also shown the nature of variation of a derived quantity, exp(γ_*T*_)/σ_*T*_, as a function of *G*_*si*_. It can potentially be used as a statistical indicator for the onset of certain types of arrhythmia that may be hard to detect by observing the skewness alone. We see from Figure 3D that the measure amplifies the signal indicating a transition close to *G*_*si*_ = 0.025 where the spiral begins to noticeably meander.

When we observe the corresponding moments for the distribution of leading digits *i* as a function of *G*_*si*_, we note that both the moments μ_*i*_ and σ_*i*_ (Figures 3E,F) have very similar nature of variation, viz., both exhibit dips around the values of *G*_*si*_ at which the different dynamical transitions occur. In contrast, the skewness exhibits an almost opposite nature, with peaks occurring at the transition points (consistent with increasing skewness of the *T* distribution at these values). This indicates that at these points the distribution comes close to the form expected from BL, as the latter is associated with positively skewed distributions. The derived quantity exp(γ_*i*_)/σ_*i*_ conserves this pattern, showing increased values at these points. We find that the skewness of *T* and that of *i* are correlated, the linear correlation coefficient between γ_*T*_ and γ_*i*_ being *r*_γ_ = 0.67 (*p* = 0.001). This indicates an inter-dependence between the variations in skewness of the time-interval distribution and that of the leading digit distribution, that occur at different dynamical transitions.

**Figure 4:**
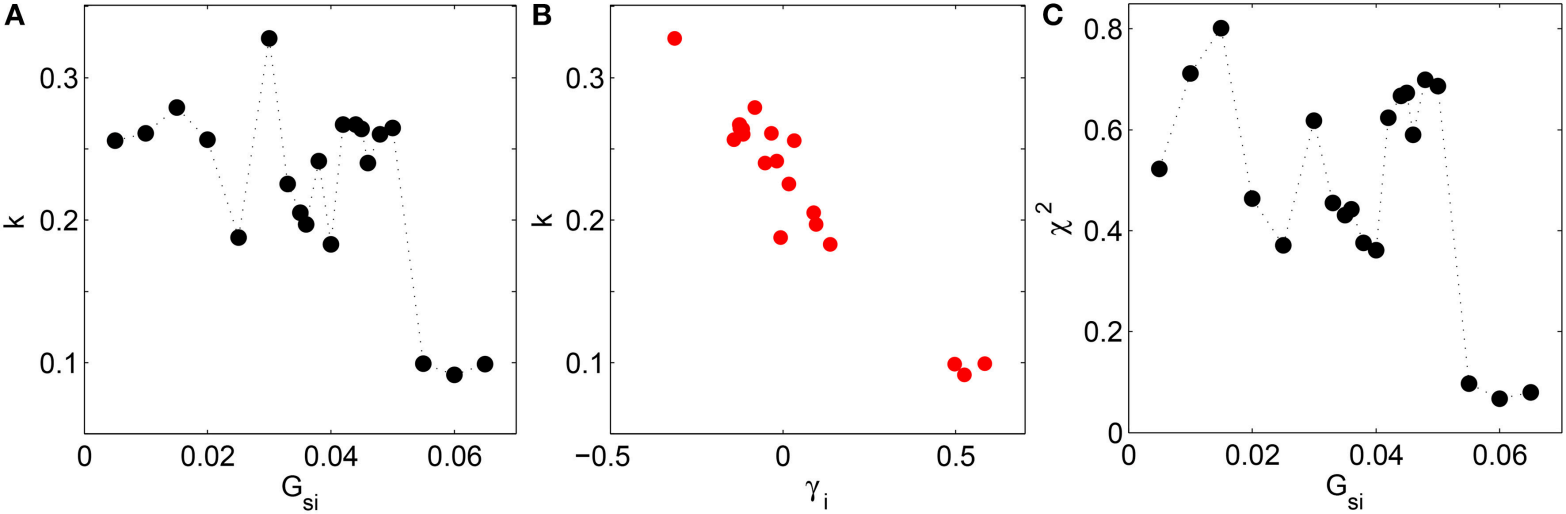
**(A)** Deviation of the distribution of leading digits *i* of the normalized time intervals *t* from Benford's law in the two-dimensional LR1 model measured in terms of the Kolmogorov-Smirnov test statistic *k* and shown as a function of the maximum Ca^2+^ channel conductance *G*_*si*_. **(B)** There is a strong negative correlation (*r* = −0.96, *p* = 10^−12^) between *k* and the skewness of the leading digits γ_*i*_. **(C)** The error in using BL for describing the empirical distribution of leading digits *i* of the normalized time intervals *t* is measured using Pearson's chi-squared test and shown as a function of *G*_*si*_. Agreement with BL improves whenever there is a dynamical transition, as seen by dips in *k* and the χ^2^ test statistic for values of *G*_*si*_ where successive transitions between rigid rotation, quasiperiodic meandering and chaotic meandering of the spiral core and spatiotemporal chaos occur.

To quantify how closely the system obeys BL in the different dynamical regimes, we show the results of different statistical tests for goodness of fit between the empirical and Benford distributions. Figure 4A shows the Kolmogorov-Smirnov (KS) test statistic as a function of the Ca^2+^ channel conductance *G*_*si*_ which clearly indicates that the distribution of leading digits follow BL most closely, indicated by dips in the test statistic, at the values of *G*_*si*_ characterizing the different dynamical transitions, viz., *G*_*si*_ = 0.025, 0.04, and 0.055 (*p*-values for the statistic are effectively zero for all *G*_*si*_). Note that low values of the KS test statistic (i.e., better agreement with BL) are associated with high positive skewness of the distribution of leading digits of the normalized intervals. This is underlined by the strong negative correlation between the test statistic *k* and the skewness γ_*i*_ (see Figure 4B), having a linear correlation coefficient *r* = −0.96 (*p* = 10^−12^). As mentioned earlier, this is consistent with the fact that BL is associated with distributions having high positive skewness. Figure 4C shows the result of another statistical test, viz., Pearson's Chi-squared test, with the lowest values of Pearson's error—corresponding to better agreement with BL—occurring at the same values of *G*_*si*_ where the dynamical transitions occur. The points at which the empirical distribution best matches BL are seen to be consistent for the two tests (the dips of the two test statistics occurring at the same values).

**Figure 5:**
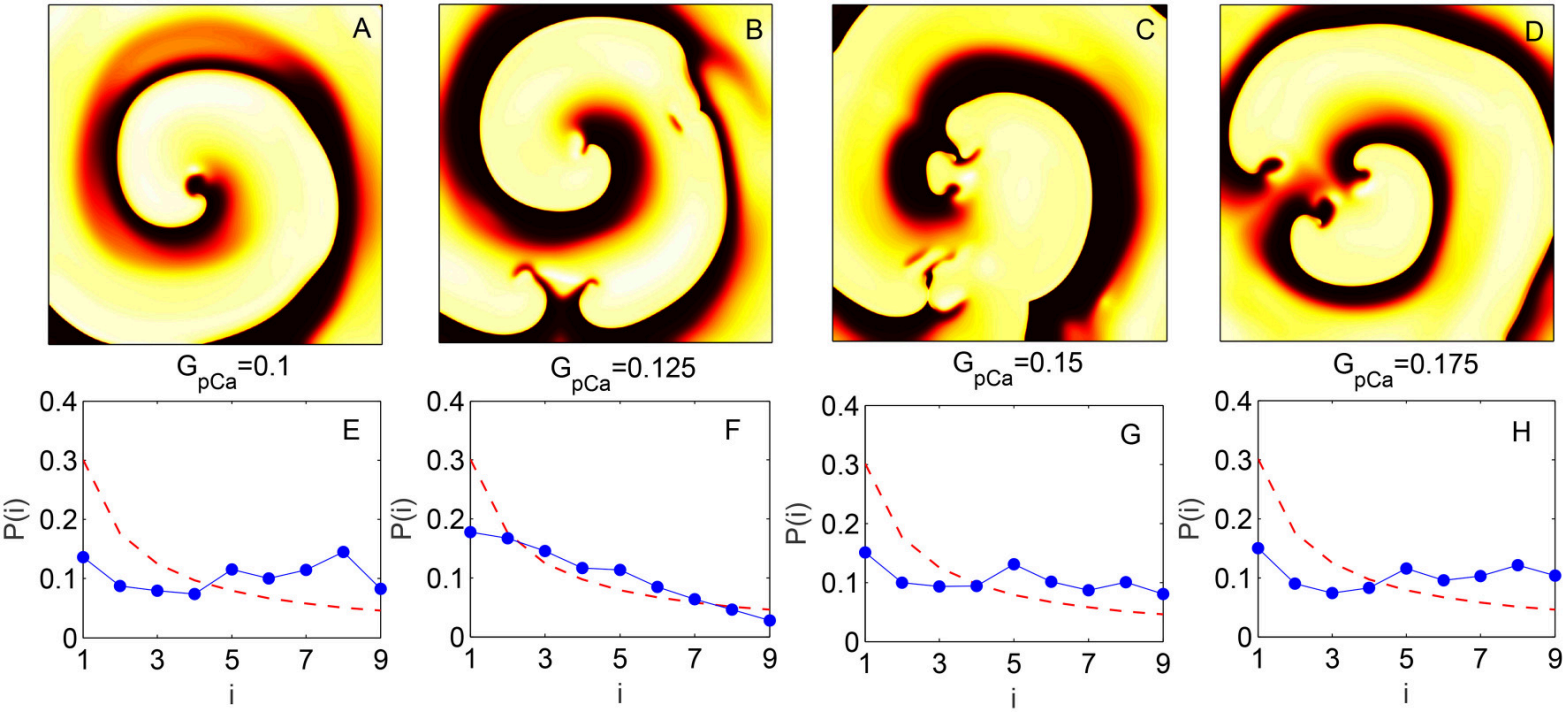
**(A–D)** Pseudocolor images of spatiotemporal activity (measured in terms of transmembrane potential *V*) for the two-dimensional TP06 model (*L* = 1024) showing the different dynamical regimes obtained by increasing the maximum conductance *G*_*pCa*_ of the sarcolemmal pump current (expressed in units of nS pF^−1^). The successive panels represent a single spiral wave at *G*_*pCa*_ = 0.1 **(A)**, the initiation of spiral breakup at *G*_*pCa*_ = 0.125 **(B)** and successive states leading to spatiotemporal chaos at *G*_*pCa*_ = 0.15 **(C)** and *G*_*pCa*_ = 0.175 **(D)**. **(E–H)** Probability distribution of the leading digits *i* of the normalized time intervals between successive supra-threshold activations of a local region corresponding to the dynamical regimes indicated in **(A–D)**, respectively. The broken curve indicates the distribution according to Benford's law. The analysis shown in **(E–H)** is performed for data obtained from a grid of 225 equally spaced points in the two-dimensional medium.

**Figure 6:**
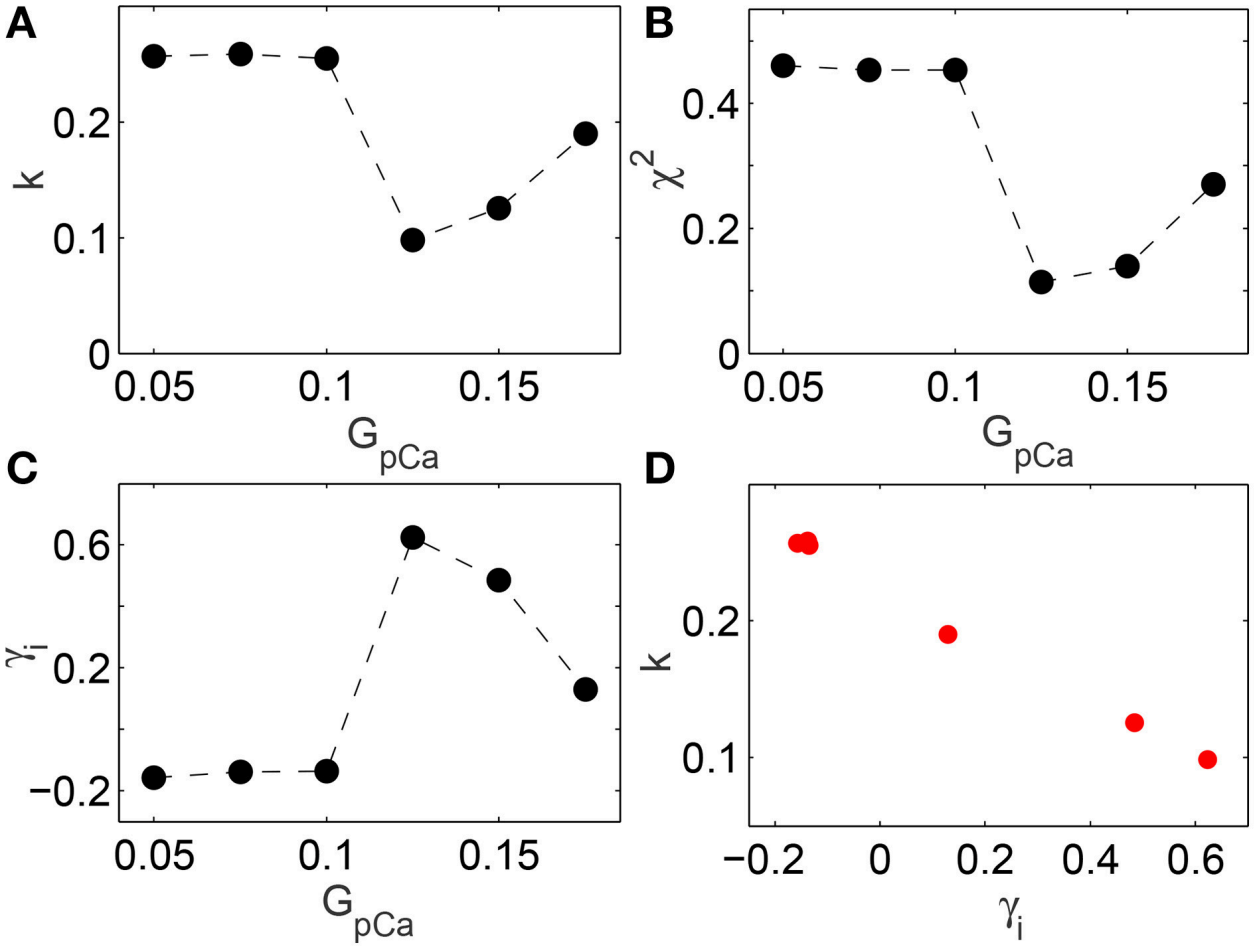
**(A,B)** Deviation of the distribution of leading digits *i* of the normalized time intervals *t* from BL in the two-dimensional TP06 model measured in terms of **(A)** the Kolmogorov-Smirnov test statistic *k* and **(B)** Pearson's Chi-squared test statistic χ^2^, shown as a function of the maximum conductance *G*_*pCa*_ of the sarcolemmal pump current (expressed in units of nS pF^−1^). Agreement with BL is highest around the value of *G*_*pCa*_ = 0.125 where breakup of the spiral wave is initiated, as seen by dips in *k* and the χ^2^ test statistic. **(C)** Skewness γ_*i*_ in the distribution of the leading digits of the normalized time interval, with the peak occurring at *G*_*pCa*_ = 0.125 corresponding to the spiral breakup transition point. **(D)** There is a strong negative correlation (*r* = −0.99, *p* = 10^−7^) between *k* and the skewness of the leading digits γ_*i*_.

The change in skewness and the agreement with BL around the transition to spiral breakup shown here do not appear to be model specific. We have explicitly verified that qualitatively similar results can be obtained by using the TP06 model for a human ventricular cell. The top row of Figure 5 shows images representative of the spatiotemporal dynamics of a two-dimensional medium in different regimes as the maximum value of conductance *G*_*pCa*_ of the sarcolemmal pump current *I*_*pCa*_ is increased. For small values of *G*_*pCa*_ a single spiral wave is seen to rotate stably in the medium, until around *G*_*pCa*_ = 0.125 nS pF^−1^ it undergoes breakup and degenerates into spatiotemporal chaos for higher values of *G*_*pCa*_. The panels in the bottom row show the distributions of the leading digits for the normalized intervals at the corresponding values of *G*_*pCa*_ indicating that the fit with Benford distribution is closest during the dynamical transitions. This is rigorously shown in Figure 6 where the results of different statistical tests for goodness of fit are given. Figures 6A,B show that both the KS and Pearson's Chi-squared tests point to a better agreement with BL at the transition point *G*_*pCa*_ = 0.125 nS pF^−1^. As with the results for LR1 model reported earlier, the transition is also associated with a large deviation in the skewness γ_*i*_ (Figure 6C) and a strong negative correlation between the test statistic *k* and the skewness (Figure 6D) with a linear correlation coefficient *r* = −0.99 (*p* = 10^−6^).

To summarize the results, around the parameter values where the transitions between dynamical regimes representative of different types of cardiac arrhythmia occur, we observe both higher positive skewness and closer agreement with BL (as indicated by statistical tests). We note that both increased skewness and better match with BL have independently been suggested earlier as possible signatures for dynamical transitions, although not in the context of physiology or clinical applications. Apart from the potential utility of this observation for devising robust indicators of the onset of life-threatening disturbances in the cardiac rhythm, it suggests a deep relation between the appearance of BL in natural phenomena and the degree of skewness in the distributions of the underlying variables.

## 4. Discussion

Statistical analysis of data (in particular, ECG) that is representative of cardiac functionality can provide us with effective signatures for the detection of arrhythmia at an early stage. Despite such analyses, certain kinds of arrhythmia fail to get detected merely due to the complexity involved. Part of the difficulty lies in cardiovascular activity being a joint outcome of intrinsic spatiotemporal excitation dynamics in heart muscle and modulation of the sinus node by the sympathetic and parasympathetic nervous system. Here we have studied biophysically detailed models of ventricular activity to infer signatures of dynamical transitions characterizing the onset of different kinds of arrhythmia. This makes it possible to disentangle the effects of intrinsic excitation dynamics in the heart from the influence of the nervous system. In principle, it allows identification of patterns that may alert one to impending harmful disruptions in the rhythmic activity of the heart, but which could be masked in the ECG signal by autonomic modulation effects. Our results show that as the spatiotemporal excitations in the ventricles become more disordered, leading to phenomena identified with sustained monomorphic and polymorphic tachycardia as well as the onset of fibrillation, these transitions are marked by characteristic changes in statistical moments associated with the distribution of inter-activation intervals. In addition, the leading digits of these intervals show a closer agreement with BL at the transition points. Our result can potentially be applied in augmenting algorithms used in implanted devices (ICDs) for detecting transitions to possible life-threatening arrhythmia so as to initiate a program of treatment (Schuckers, 1998). Thus, when continual monitoring of heartbeat time-series shows either increased skewness or a closer agreement to BL, it may signal a transition in the dynamical state of the heart so that suitable pacing therapy can be started. However, for such an application, our observations would need to be validated in ECG recordings made during tachycardia and onset of fibrillation in live animals. Such validation is necessary in view of the limitations of the present study, involving as it does two-dimensional monodomain models of homogeneous cardiac tissue.

Examining the passage from normal cardiac activity to different arrhythmic regimes from the perspective of phase transitions can provide novel insights, as has been pointed out by several earlier studies. For instance, power-law behavior, which characterize critical phenomena in physical systems, have been reported in R-R interval fluctuations and are seen to be remarkable predictors of arrhythmic death, with a steeper negative slope of the power spectra (in log scale) clearly distinguishing a diseased heart from a healthy one (Bigger et al., 1996). More recently, it has been shown that phase transition-like dynamics is exhibited by healthy human heart rate, indicated by long range correlations which is a hallmark of criticality (Kiyono et al., 2005). In contrast, the dynamics of an abnormal heart rate reveals significant digression from critical behavior. In addition, scale invariance, which is seen in systems close to critical point, has been shown to be indicative of a healthy heart—with its absence being a statistical feature that can alert us about pathological conditions (Kiyono et al., 2004). Our results provide yet another connection between onset of arrhythmia and phase transitions by showing that sharp changes in the skewness of the distribution of dynamical variables, that has previously been associated with dynamical transitions in other systems (Guttal and Jayaprakash, 2008; Scheffer et al., 2009), can potentially act as a robust indicator of transitions between monomorphic tachycardia, polymorphic tachycardia and fibrillation in the heart.

As mentioned earlier, the appearance of BL has also been linked to phase transitions in physical systems (De and Sen, 2011). As the Benford distribution follows from requirement of scale invariance of the underlying numbers (Hill, 1995), it is tempting to connect this with the scale invariance of distribution of dynamical variables associated with critical points at which transitions occur. We observe from our results that parameter regimes that give rise to relatively broad distributions of the time-intervals (as is the situation for spatiotemporally chaotic states) show better agreement with BL than those associated with highly confined distributions (as in the case of a rigidly rotating spiral). However, the occurrence of chaos in dynamical systems by itself does not guarantee that BL will be obeyed (Tolle et al., 2000). Thus, it appears that the appearance of Benford distribution is more closely associated with the onset of dynamical transitions rather than the specific nature of the dynamical states on either side of the transition point. We also note that distributions that follow BL are, in general, associated with high skewness (Scott and Fasli, 2001). This suggests that the highly skewed nature of distributions during dynamical transitions and the observation of better agreement with BL at those points may not be independent of each other. Thus, our results imply that the increased skewness associated with regime shifts and the appearance of Benford distribution during phase transitions—which have been reported earlier in different contexts—are, in fact, related.

### Conflict of interest statement

The authors declare that the research was conducted in the absence of any commercial or financial relationships that could be construed as a potential conflict of interest.
